# From granulomas to tumors: post-tuberculosis immune and structural lung remodeling as a driver of carcinogenesis

**DOI:** 10.3389/fimmu.2026.1889276

**Published:** 2026-08-19

**Authors:** Praise Audax Rukonge, Vincent Kawuribi, Zhimeng Lu, Adelaiye Favour Oluwaseun, Yifan Sheng, Kun Yan, Zhiyang Zheng, Xinyao Liu, Liang Chu, Guiping Yu

**Affiliations:** 1 Department of Cardiothoracic Surgery, Jiangyin Clinical College of Xuzhou Medical University, Wuxi, Jiangsu, China; 2 Department of Biomedical Engineering, School of Medical Imaging, Xuzhou Medical University, Xuzhou, China; 3 Department of Cardiothoracic Surgery, The Affiliated Jiangyin Hospital of Nantong University, Jiangsu, China; 4 The Affiliated Hospital of Henan University of Science and Technology, Luoyang, China; 5 Wuxi Key Laboratory of Biomaterials for Clinical Application, Department of Central Laboratory, Jiangyin Clinical College of Xuzhou Medical University, Wuxi, China; 6 Department of Cardiothoracic Surgery, The Affiliated Hospital of Xuzhou Medical University, Xuzhou, Jiangsu, China; 7 Department of Thoracic Surgery, Shanghai Public Health Clinical Center (Fudan University), Shanghai, China

**Keywords:** immune exhaustion, lung cancer, post-tuberculosis sequelae, tuberculosis, tumor microenvironment and carcinogenesis

## Abstract

Although antibiotic therapy effectively cures active tuberculosis (TB), many survivors are left with permanent lung damage and long-lasting immune alterations. Growing epidemiological evidence indicates that individuals with prior pulmonary TB have a two- to three-fold increased risk of lung cancer, independent of smoking, suggesting mechanisms beyond shared risk factors. This review advances the concept that TB imprints a durable “memory” within the lung, characterized by persistent structural remodeling and immune reprogramming that together create a tumor-permissive microenvironment. We synthesize evidence showing that TB granulomas act as dynamic immune niches that induce hypoxia, fibrosis, and immune exhaustion, features that frequently persist after microbiological cure. Post-TB sequelae including fibrotic scarring, cavitation, bronchiectasis, and vascular remodeling, promote chronic inflammation, oxidative DNA damage, and mechanotransduction pathways linked to oncogenesis. Concurrently, sustained T-cell exhaustion, macrophage polarization toward tumor-associated phenotypes, and impaired antigen presentation weaken tumor surveillance. We further discuss emerging roles for lung microbiome dysbiosis in sustaining inflammation. Collectively, these processes provide a mechanistic framework linking healed TB to lung carcinogenesis and highlight TB survivors as a distinct population for targeted surveillance and preventive strategies.

## Graphical Abstract

**Figure:**
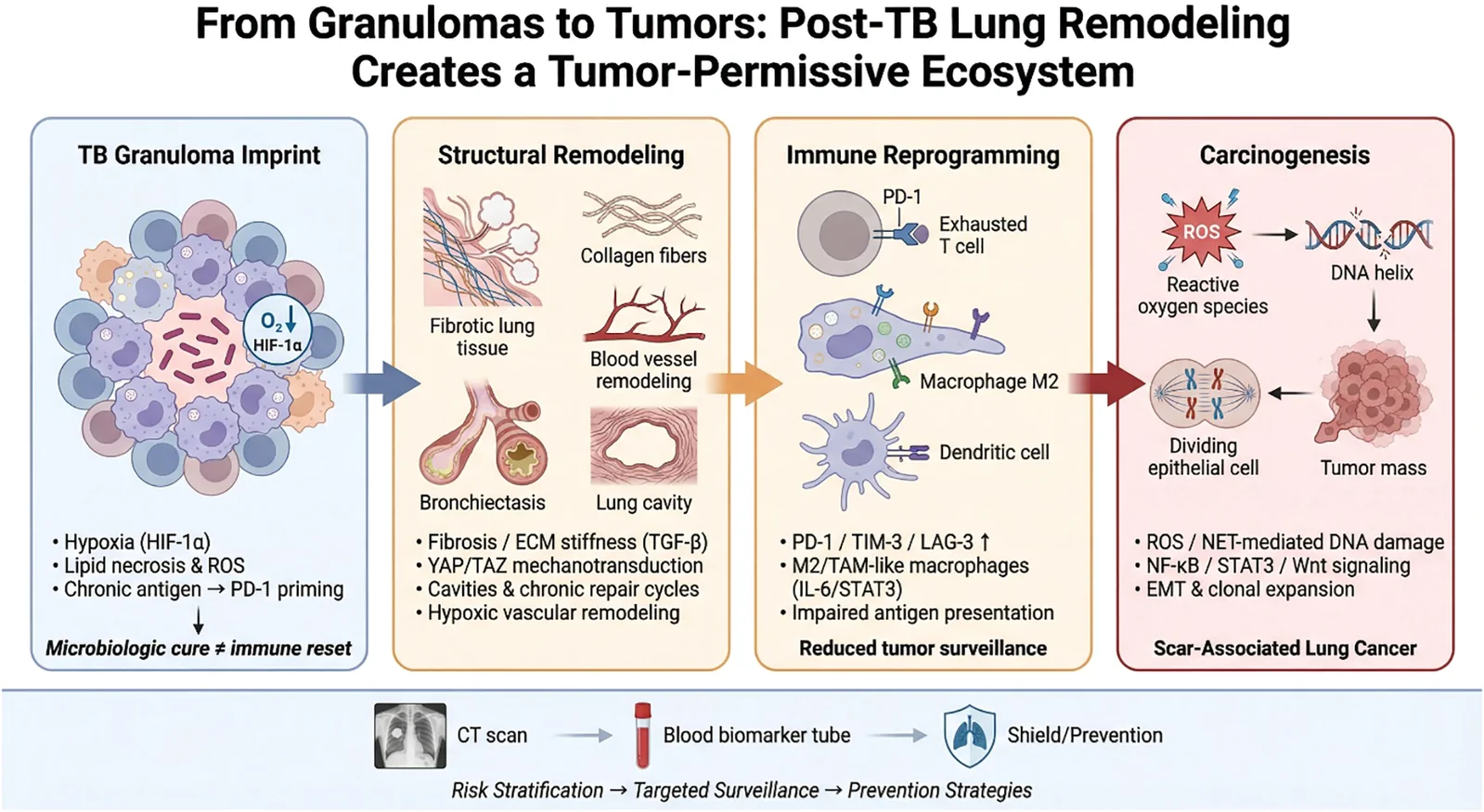
Pulmonary tuberculosis leaves a durable structural and immune imprint within the lung. Even after microbiologic cure, residual granuloma-derived hypoxia, fibrosis, cavitation, and vascular remodeling create a chronically inflamed and mechanically altered tissue scaffold. Concurrent T-cell exhaustion, macrophage reprogramming, and impaired antigen presentation weaken tumor surveillance. Persistent ROS generation and NF-κB/STAT3/Wnt signaling promote DNA damage, epithelial–mesenchymal transition, and clonal expansion, ultimately predisposing to scar-associated lung cancer. This framework positions TB survivors as a biologically distinct high-risk population for targeted surveillance and prevention.

## 1 Introduction: TB Is cured but the lung is not

**Figure 1:**
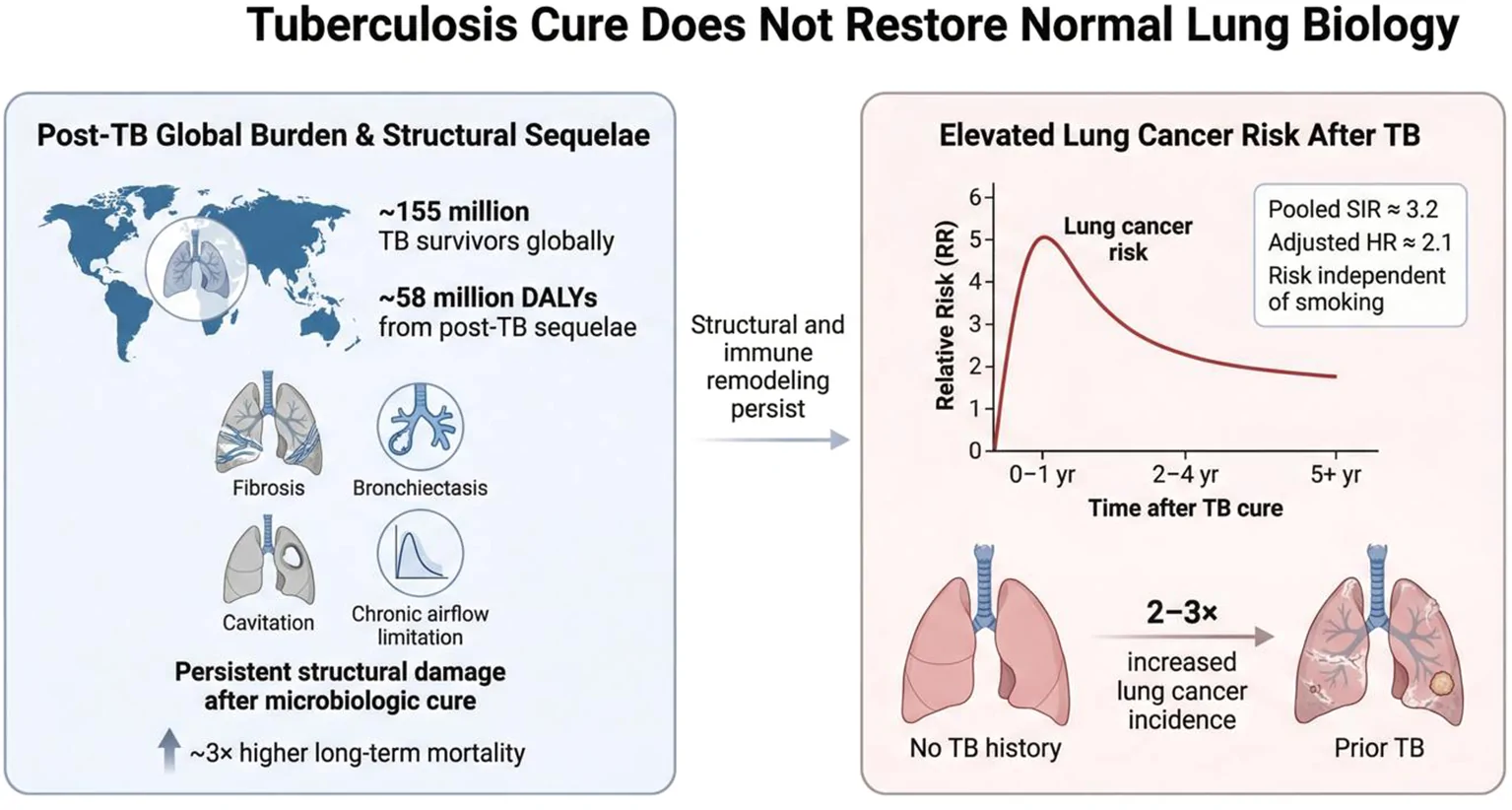
Tuberculosis cure does not restore normal lung biology and is associated with increased lung cancer risk. Despite microbiologic eradication, an estimated 155 million TB survivors globally experience persistent structural sequelae including fibrosis, cavitation, bronchiectasis, and airflow limitation, contributing substantially to long-term morbidity and mortality. Epidemiologic studies consistently demonstrate a 2–3-fold increased incidence of lung cancer following pulmonary TB, with the highest risk observed in the first years after cure but persisting long-term, independent of smoking. These observations suggest that TB leaves a durable structural and immunologic imprint that predisposes to carcinogenesis. Created with Biorender.com.

Although effective antibiotic therapy “cures” active tuberculosis (TB), the lungs of survivors often remain chronically damaged. An estimated 155 million people have survived TB globally (1), and studies suggest up to half of these survivors suffer long-term pulmonary sequelae (2, 3). Indeed, nearly half of TB’s global health burden (58 million DALYs) is now attributed to post-TB sequelae (3). Common long-term manifestations include reduced airflow, fibrosis, bronchiectasis and cavitary lesions (**Figure 1**) (4–7). These structural changes produce chronic respiratory symptoms (cough, dyspnea) and elevate mortality (standardized mortality ~3× higher than general population (8), even many years after microbiological cure.

Concurrently, epidemiology is revealing a concerning pattern: TB survivors have a markedly increased risk of lung cancer, independent of smoking. Multiple meta-analyses now report roughly a 2–3-fold elevation in lung cancer incidence among people with prior TB (9, 10). For example, the systematic review by *Luczynski* et al. (9) reported a pooled standardized incidence ratio (SIR) of ~3.2 and adjusted hazard ratio (aHR) ~2.1 for lung cancer following TB (9). Notably, this risk is highest in the first 1–2 years after TB but remains modestly elevated even 5+ years later (9, 10). The effect persists in never-smokers (9, 11) and in diverse settings. In one large cohort study, TB was an independent lung cancer risk factor regardless of smoking or chronic obstructive pulmonary disease (COPD) status (11). Taken together, these signals suggest the association is not merely confounding by shared risk factors but may be causal or at least permissive.

Historically, the architectural relationship between chronic tissue destruction and oncogenesis has been recognized since Friedrich first described ‘scar carcinoma’ in 1939 (12). Classical pathology viewed tuberculous scars may serve as sites of carcinogenesis (13, 14), molecular pathology reveals that both active lesions and healed post-tuberculosis (TB) scars are dynamic, pro-tumorigenic microenvironments (15, 16).Non-small cell lung cancer (NSCLC), which accounts for 85% of all lung cancers, is the histological subtype that is most frequently associated with previous TB (17, 18). The histopathological manifestation diverges by lesion status: inactive, fibrotic scars frequently host lung adenocarcinoma (LUAD) in the peripheral upper lobes, which is the predominant subtype identified in post-TB lungs, whereas active, chronic cavitary lesions are more commonly associated with central squamous cell carcinoma (SCC) driven by persistent bronchial irritation (14, 19). Recent clinical data comparing patients with coexisting pulmonary TB and lung cancer with patient with lung cancer alone have suggested that TB-associated lung cancer may present with more advanced pathological characteristics, including increased lymph node involvement and distant metastasis (20).The primary evolutionary origin of these cancers is rooted in the long-term cellular transitions of localized alveolar epithelial cells (AECs), including alveolar type II (AT2), club cells and bronchioalveolar progenitor cells which may also contribute to carcinogenesis under specific genetic or inflammatory conditions (21, 22). Recent studies further indicate that TB-associated lung cancers differ from biologically smoking-related lung cancers. Smoking-related lung cancers exhibit a high tumor mutational burden (TMB) and heavy TP53 mutations; this is due to the fact that tobacco smoke promotes carcinogenesis through the accumulation of DNA mutations induced by chemical carcinogens (23). While TB-driven NSCLC closely mimics a ‘never-smoker’ profile, demonstrating a significantly higher prevalence of epidermal growth factor receptor (EGFR) driver mutations and is associated with worse treatment responses to EGFR tyrosine kinase inhibitors (TKIs) (24, 25).

These observations motivate a central thesis: TB leaves a durable “memory” in the lung, embedding both immune and structural alterations that prime the tissue for malignant transformation. We propose that chronic TB infection imprints a persistent state of immune dysregulation and tissue remodeling. A tumor-permissive microenvironment, even after the bacteria are eliminated. The mechanistic basis for positioning tuberculosis (TB) as a long-term architect of lung-cancer risk emerges from converging epidemiologic and biological evidence demonstrating that chronic pulmonary infection can initiate and sustain carcinogenic processes. This association aligns with a broader paradigm in which persistent infection and inflammation create a permissive microenvironment for malignant transformation (26).

To formalize this relationship, we propose a three-stage mechanistic model for TB-associated lung carcinogenesis that integrates established biological principles with emerging TB-specific observations.

### 1.1 Initiation

Granuloma-associated hypoxia, together with sustained production of reactive oxygen and nitrogen species (ROS/RNS), generates a microenvironment of oxidative stress capable of inducing DNA damage, lipid peroxidation, and genomic instability in adjacent bronchoalveolar epithelium (26, 27). Chronic inflammatory states are well established to promote mutagenesis through ROS-mediated DNA damage and error-prone repair processes, thereby initiating carcinogenesis (26, 27). In TB, these processes occur within structurally confined granulomatous lesions, producing a mutational burden that likely overlaps with, but is mechanistically distinct from, tobacco-induced polycyclic aromatic hydrocarbon (PAH) damage. While TB-specific mutational signatures remain incompletely characterized, the principle of inflammation-driven genotoxic stress is strongly supported.

### 1.2 Promotion

Following microbiologic cure, the affected lung does not revert to baseline but instead evolves into a chronically remodeled tissue state characterized by fibrosis, immune dysregulation, and persistent inflammatory signaling (28). Fibrotic remodeling and extracellular matrix (ECM) deposition are well-recognized drivers of tumorigenesis, acting through altered tissue mechanics, impaired immune surveillance, and sustained cytokine signaling (29). Central to this process are pro-tumorigenic pathways such as TGF-β and IL-6/STAT3 signaling, which promote epithelial–mesenchymal transition (EMT), survival, and proliferation of pre-malignant cells (26). STAT3 activation, in particular, represents a canonical link between chronic inflammation and cancer development, integrating cytokine signaling with transcriptional programs that favor tumor growth and immune evasion (27).

Concurrently, mechanical remodeling of the lung parenchyma results in increased matrix stiffness, a key regulator of mechanotransduction pathways such as YAP/TAZ signaling, which have been implicated in both fibrosis and oncogenesis (29). Fibrotic tissues provide a mechanically permissive niche that enhances proliferative signaling and cellular plasticity, thereby facilitating clonal expansion of initiated epithelial cells. In parallel, persistent immune activation following TB leads to long-term reprogramming of innate immune cells and the accumulation of antigen-experienced, exhausted T-cell populations. Chronic infection and inflammation are known to induce immune exhaustion phenotypes and checkpoint pathway activation, which can impair tumor immune surveillance and promote tumor escape (26).

Together, these processes define a discrete immunopathological state, here conceptualized as a post-TB pre-malignant niche, characterized by localized architectural distortion (fibrosis, cavitation, bronchiectasis), persistent inflammatory signaling, and immune exhaustion.

### 1.3 Progression

Within this niche, ongoing inflammation, fibrosis, and immune dysregulation create selective pressure favoring clonal expansion of oncogenically primed cells. Chronic inflammatory microenvironments are known to support tumor progression by promoting angiogenesis, suppressing immune responses, and facilitating metastatic potential. Notably, lung cancers arising in non-smoking populations are frequently driven by epidermal growth factor receptor (EGFR) mutations, which represent one of the dominant oncogenic pathways in this context (30). Emerging evidence suggests that chronic inflammatory and fibrotic lung conditions, such as TB-associated lung adenocarcinoma, have a higher prevalence of EGFR mutations (24)However, this staged model is mechanistically distinct from other chronic lung disease–associated carcinogenesis paradigms. In idiopathic pulmonary fibrosis (IPF), tumorigenesis is typically diffuse, basal, and frequently associated with KRAS and TP53 alterations with lower EGFR mutations (31, 32), whereas in COPD, carcinogenesis is closely linked to smoking-induced mutational signatures and centrilobular emphysema. By contrast, TB-associated carcinogenesis is spatially focal, granuloma-derived, and often localized to structurally altered segments of lung parenchyma, reflecting the site-specific nature of prior infection.

Importantly, this framework generates testable biological predictions. Post-TB lesions should demonstrate persistent inflammatory signaling, immune exhaustion signatures, and fibrotic remodeling exceeding that of unaffected lung tissue. Furthermore, tumors arising in this context may exhibit distinct immunologic and molecular characteristics, including differential responses to immune checkpoint blockade compared with smoking-driven cancers.

To operationalize this concept for clinical and translational investigation, we propose the term Post-TB Pre-Malignant Immunological Scar (PTPMIS), defined as a measurable phenotype integrating prior TB exposure, residual structural lung disease, persistent inflammation, and immune dysregulation. While the specific thresholds for biomarkers and imaging parameters remain to be validated, each component is individually supported by existing evidence linking chronic inflammation, fibrosis, and immune dysfunction to carcinogenesis.

Finally, the TB–lung cancer axis aligns with a broader and well-established infection-to-cancer paradigm, exemplified by hepatitis C virus–associated hepatocellular carcinoma, *Helicobacter pylori*–associated gastric cancer, and inflammatory bowel disease–associated colorectal cancer. In each case, chronic inflammation, tissue remodeling, and immune dysregulation precede malignant transformation. TB-associated carcinogenesis fits within this conceptual framework while remaining mechanistically distinct in its granuloma-driven, spatially localized, and fibrosis-dominated biology. In this view, prior TB is not just a resolved infection, but a lifelong architect of lung cancer risk.

TB cure does not fully restore normal lung biology. Millions of TB survivors suffer chronic lung disease (3). These sequelae include fibrosis, bronchiectasis, cavities and immune scars (4–6). Epidemiological studies consistently find elevated lung cancer incidence years after TB (9, 11). In the sections below, we explore how granulomas and chronic TB immunity sow the seeds of cancer from fibrotic scar “soil” to exhausted immune “guards”, intersecting with classical oncogenic pathways.

## 2 Granuloma biology: the origin of long-term remodeling

**Figure 2:**
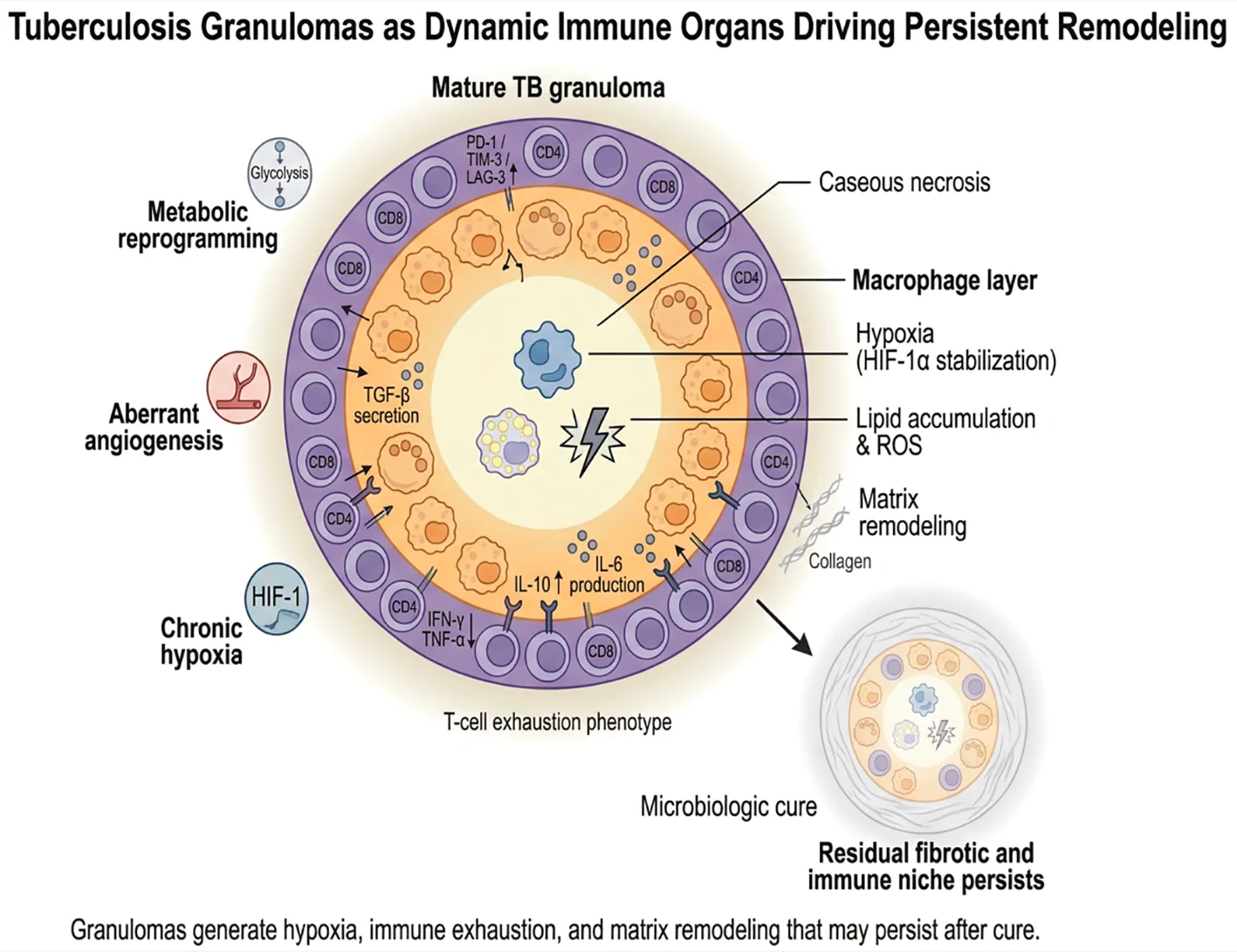
Tuberculosis granulomas function as dynamic immune organs that generate persistent remodeling signals. Active granulomas contain hypoxic, necrotic cores enriched in lipid-laden macrophages and reactive oxygen species, surrounded by macrophage and lymphocyte layers. Chronic antigen stimulation drives T-cell exhaustion marked by PD-1, TIM-3, and LAG-3 upregulation with increased IL-10 and reduced effector cytokines. Granuloma-associated cytokines and metabolic stress promote fibrosis, angiogenesis, and tissue remodeling. Even after microbiologic cure, residual fibrotic and immune niches may persist, establishing a foundation for long-term structural and immune dysregulation. Created with Biorender.com.

TB granulomas are not inert scars but dynamic immune microenvironments (28). In active pulmonary TB, infected macrophages aggregate into organized lesions with a necrotic, caseous core and fibrotic rim (33). These structures comprise a mix of cell types: epithelioid macrophages, foamy lipid-laden macrophages, multinucleated giant cells, and infiltrating lymphocytes (33). Importantly, granulomas evolve over time as the host and pathogen interact, they expand, contract, calcify or cavitate, reflecting ongoing immune activity rather than forming static scars (**Figure 2**).

Within granulomas, the microenvironment is profoundly altered. Hypoxia is common: TB granulomas in animal models and humans develop oxygen-deprived cores (34). PET imaging shows heterogeneous hypoxic zones around cavities (34). Hypoxia stabilizes HIF-1α in macrophages and epithelial cells, driving glycolytic metabolism and pro-survival signaling (35–37). Necrosis and lipid accumulation also occur: foamy macrophages filled with lipids accumulate near necrotic centers, both a hallmark of TB and a source of oxidative stress (38–41). Together hypoxia, necrosis and associated reactive species create metabolic stress on local cells.

Crucially, the immune cells in chronic granulomas show signs of exhaustion. T-cells initially enter granulomas in a highly activated state, but with persistent antigen they upregulate inhibitory receptors. In late-stage TB lesions, CD4^+^ and CD8^+^ T cells co-express PD-1, TIM-3 and LAG-3 (42, 43), and secrete high IL-10 but low IFN-γ/TNFα (44). In other words, chronic TB drives T-cell exhaustion even before cure. Experimental TB in mice confirms that knockout of PD-1/PD-L1 yields uncontrolled immunity, implying that wild-type TB actively exploits PD-1 to dampen T cells (45). Thus, by the end of infection, many T cells in granulomas have an “exhausted” phenotype typical of chronic viral infections or tumors.

Granuloma resolution is often incomplete. Even after antibiotics clear the bacilli, most granulomas do not fully revert to normal lung. Residual architectural scars areas of fibrosis and calcification are left behind (28). Critically, the immune milieu remains imbalanced. For example, “resolved” granulomas can persist as nodules containing persistent lymphocytes and macrophages with aberrant activation states (46). Hypoxic, fibrotic niches created by prior lesions may remain chronically hypoxic and inflamed. In sum, granulomas seed the lung with patches of dysregulated immunity, chronic inflammation, and structural damage. These persistent scars set the stage for later carcinogenesis (see below).

## 3 Structural sequelae of TB: building a pro-tumor scaffold

Tuberculosis often leaves behind physical lung damage that itself can promote cancer. We consider three major sequelae: fibrosis/scarring, cavitation/bronchiectasis, and vascular remodeling. Each creates a microenvironment conducive to malignancy as summarized in **Table 1**.

**Table 1:** Post-tuberculosis sequelae and pro-carcinogenic mechanisms.

TB sequela	Pathological features	Pro-carcinogenic mechanisms	References
Fibrosis/Scarring	Excess extracellular matrix deposition and collagen accumulation driven by TGF-β signaling, resulting in a stiff fibrotic lung microenvironment.	ECM stiffening activates mechanotransduction pathways such as YAP/TAZ, promoting epithelial proliferation, survival signaling, and epithelial-mesenchymal transition (EMT). Persistent TGF-β signaling also induces oxidative stress and genomic instability, contributing to malignant transformation.	(29)
Bronchiectasis	Irreversible bronchial dilatation, mucus stasis, and chronic bacterial colonization.	Persistent infection drives neutrophilic inflammation characterized by cytokines (e.g., IL-8, TNF-α), reactive oxygen species (ROS), proteases, and neutrophil extracellular traps (NETs). These processes cause DNA damage, epithelial injury, and dysplasia, while chronic colonization promotes a tumor-permissive microenvironment.	(47)
Cavitation	Caseous necrosis resulting in permanent pulmonary cavities with ongoing tissue destruction.	Repeated cycles of epithelial injury and repair at cavity margins increase mutational burden. Local hypoxia within cavities activates HIF-1α signaling, which stimulates angiogenesis and metabolic reprogramming that can support tumor initiation.	(48)
Vascular Remodeling	Abnormal angiogenesis, capillary destruction, and disorganized vascular architecture.	Chronic hypoxia induces sustained HIF-1α activation, driving glycolytic metabolism and VEGF-mediated neovascularization. The resulting abnormal vasculature facilitates tumor invasion, metastatic dissemination, and further genomic instability through hypoxia-driven stress.	(48)

### 3.1 Fibrosis and scar-associated carcinogenesis

A common outcome of pulmonary TB is extensive fibrosis, excessive collagen deposition and stiffening of lung tissue (49, 50). This is driven by chronic wound healing signals: macrophages and epithelial cells in TB lesions secrete high levels of TGF-β and other fibrogenic cytokines (51). TGF-β promotes fibroblast activation, collagen synthesis, and architectural distortion (52). The result is scar tissue that is rigid and abnormally remodeled.

Fibrotic scars impose pro-carcinogenic conditions. Increased extracellular matrix (ECM) stiffness engages mechanotransduction pathways in adjacent epithelial cells (53–55). Stiff fibrotic lung can activate YAP/TAZ and integrin signaling, driving epithelial cell proliferation and survival (a known effect in idiopathic pulmonary fibrosis models). TGF-β itself is a cytokine that can have dual roles: initially anti-inflammatory, but chronically it drives epithelial–mesenchymal transition (EMT) and release of reactive oxygen species (ROS). These changes can induce DNA damage and promote a pro-invasive phenotype in epithelial cells. Clinically, the notion of “scar carcinoma” lung cancers arising in fibrotic scars has long been noted (even before it was well understood molecularly), especially in upper lung zones with old TB scars. Although mechanistic studies of scar carcinogenesis in TB are sparse, the parallels with other fibrotic lung diseases (e.g. IPF–associated lung cancer (53)) suggest a similar link. Fibrotic remodeling also creates pockets of chronic inflammation: fibroblast–immune cell cross-talk sustains low-level cytokine production (TGF-β, TNF, IL-6). Over time, this milieu fosters mutations and clonal outgrowth in nearby epithelia.

Bronchiectasis (permanent airway dilation) is another frequent aftermath of TB, often adjacent to old cavities (56). The bronchiectasis lung is prone to recurrent bacterial colonization, especially by Gram-negative bacilli (e.g. *Pseudomonas aeruginosa*) *(*57–59). These chronic infections drive neutrophil-dominated inflammation: cytokines like IL-8, IL-1β, TNF and LTB4 attract and activate neutrophils (47). Activated neutrophils release myeloperoxidase, elastase, and reactive oxygen/nitrogen species (ROS/RNS), and even form neutrophil extracellular traps (NETs) (60–62). Such factors are directly genotoxic to epithelial cells, causing DNA strand breaks and mutations during the repeated injury repair cycles in bronchiectasis airways. Bronchial epithelial cells in these niches are thus under intense replication stress. Over decades, this likely increases the probability of oncogenic mutations. In sum, both cavities and bronchiectasis provide persistent sources of injury of epithelial cells continuously regenerate under inflammatory stress, creating replication-linked DNA damage and promoting tumor-promoting signals.

Finally, TB induces vascular and hypoxic remodeling. Granulomas stimulate angiogenesis (often disorganized), but many vessels later regress, leaving regions of chronic hypoperfusion. Elevated HIF-1α signaling, as seen in hypoxic granulomas (35), induces metabolic reprogramming: epithelial and stromal cells shift toward glycolysis and lactate production. These metabolic changes favor survival in hypoxia but can also select for cells with oncogenic adaptations (e.g. upregulated Glut1, LDHA). Moreover, chronic hypoxia perpetuates inflammation (via NF-κB) and limits immune cell function. Aberrant vasculature and hypoxic niches are hallmarks of the tumor microenvironment. In TB-damaged lung, similar features of patchy vascular remodeling, leaky vessels, and persistent HIF-1α activity are likely, sustaining a microenvironment that encourages malignant transformation.

**Figure 3:**
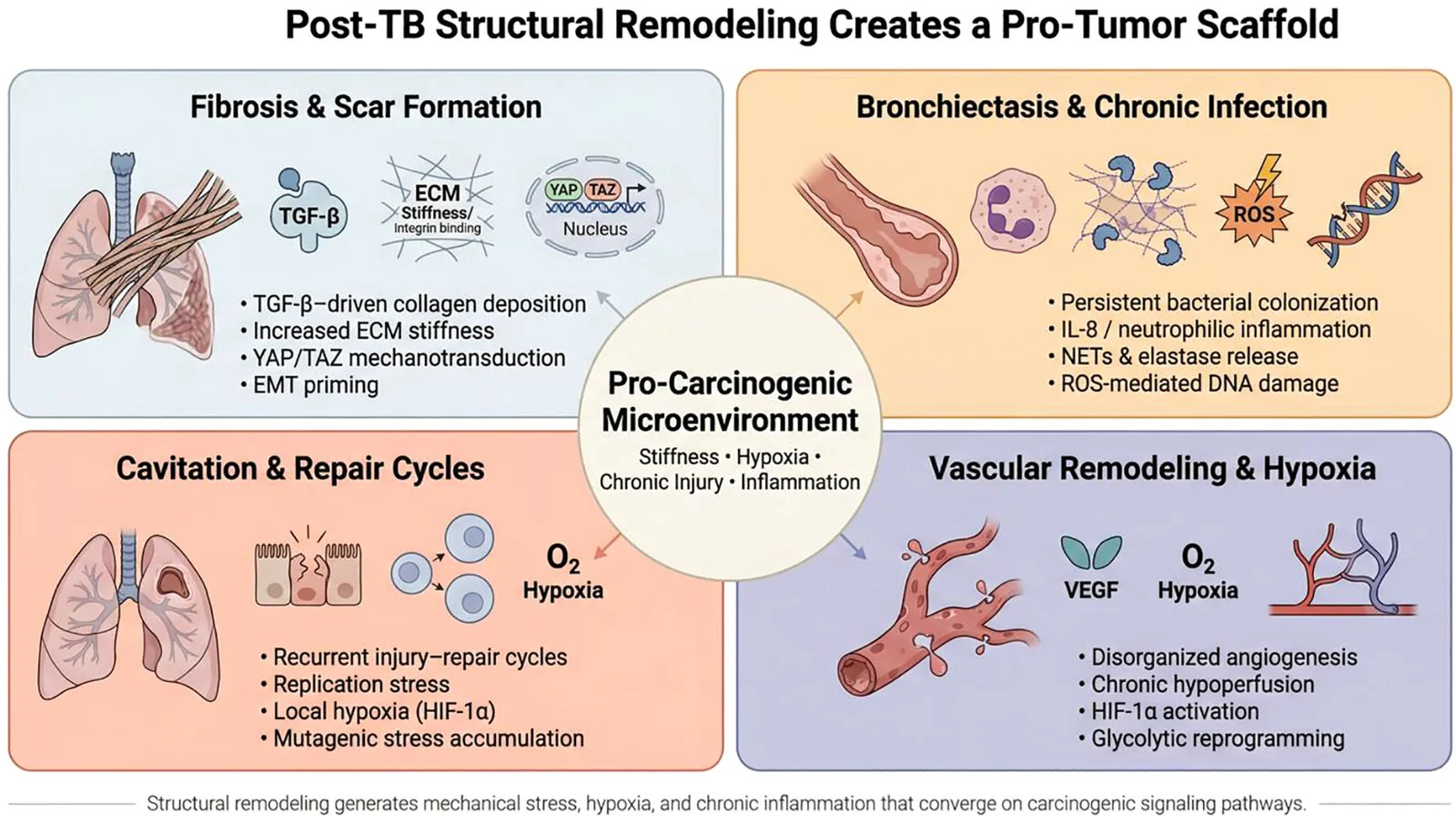
Post-tuberculosis structural sequelae create a pro-carcinogenic scaffold. Fibrosis driven by TGF-β results in extracellular matrix stiffening and YAP/TAZ-mediated mechanotransduction. Bronchiectasis sustains chronic bacterial colonization and neutrophil-mediated oxidative DNA damage. Cavitary lesions promote recurrent epithelial injury–repair cycles and replication stress. Vascular remodeling and hypoperfusion reinforce chronic hypoxia and angiogenic signaling. Together, these structural alterations generate a mechanically and metabolically altered lung microenvironment that favors malignant transformation. Created with Biorender.com.

In summary, TB scars the lung as a “soil” for cancer: excessive fibrosis, chronic cavities/bronchiectasis, and hypoxic vasculature together create a milieu of stiffness, oxidative stress, and inflammation that promotes carcinogenesis (**Figure 3****).**

## 4 Post-TB immune reprogramming: loss of tumor surveillance

Tuberculosis not only scars the lung but profoundly reprograms immunity. After TB cure, the host does not simply return to a “baseline” state instead, the immune landscape remains altered in ways that favor tumor evasion. This section details three axes of immune reprogramming: exhausted T cells, macrophage polarization, and impaired antigen presentation.

### 4.1 T-cell exhaustion and immune checkpoint upregulation

During chronic TB, sustained antigenic stimulation drives CD4^+^ and CD8^+^ T cells into an exhausted phenotype. Key inhibitory receptors become persistently upregulated: TB granuloma T cells express high PD-1, TIM-3 and LAG-3 (43, 63). For example, in late TB infection, a CD4^+^ subset co-expresses TIM-3 and PD-1 along with LAG-3, accompanied by elevated IL-10 and suppressed IFN-γ/TNF-α production (44)These cells have markedly reduced cytotoxicity and cytokine secretion. Even after antibiotic cure, some TB-specific T cells remain phenotypically exhausted. Studies of TB patients show that PD-1/PD-L1 levels on circulating T cells and monocytes are abnormally high (64–66), indicating chronic checkpoint engagement.

Such exhaustion likely weakens tumor surveillance. Cytotoxic CD8^+^ T cells that control nascent tumor clones may remain hypofunctional if their PD-1–CTLA-4 axes are already engaged by prior TB. Indeed, blocking PD-1 in animal models can reverse TB-induced T-cell dysfunction (67), but similar exhaustion might dampen the response to emerging tumor antigens. Moreover, TB-induced PD-L1 expression is not limited to T cells: infected macrophages and other cells upregulate PD-L1, further quenching effector T cells (68). The net effect is a T-cell compartment that is skewed away from effective cytotoxic and helper responses and toward immunoregulation. In a post-TB lung, this could mean that early cancer cells encounter a less vigilant T-cell response.

### 4.2 Macrophage re-education and trained innate immunity

Monocytes and macrophages also undergo long-lasting reprogramming after TB. During infection, M. tuberculosis actively promotes an M2-like, tolerogenic macrophage phenotype. Monocytes from active TB patients show impaired differentiation into classical M1 cells and instead adopt alternative activation markers e.g. elevated CD163, MerTK and STAT3 signaling (69). *In vivo*, the outer zones of human granulomas are enriched in IL-4 type (M2-like) macrophages with high arginase activity (70). This TB-driven M2 skew suppresses bactericidal functions and favors tissue remodeling. Chronic TB is also marked by high IL-6 and IL-10 production, which further reinforce STAT3 signaling in macrophages and lymphocytes.

Crucially, these macrophages begin to resemble tumor-associated macrophages (TAMs). Both TB and tumors rely on macrophages to remodel tissue and suppress immunity. Persistent IL-6/STAT3 signaling is particularly important: TB granulomas have abundant IL-6, which activates STAT3 in incoming immune cells, driving anti-inflammatory gene expression (e.g. IL-10, Arginase-1). Such STAT3-activated macrophages mirror TAMs that support angiogenesis and inhibit T-cell activation. There is emerging evidence for epigenetic “trained immunity” effects: M. tuberculosis infection leaves marks on monocyte chromatin (e.g. altered histone methylation) that can last for months (71). These changes may blunt future innate responses or bias monocytes toward regulatory phenotypes. For example, Mtb-infected macrophages show repressive histone marks at genes for ROS production (72), limiting their antimicrobial (and potentially anti-tumor) activity. While trained immunity is usually discussed in the context of enhanced responses, Mtb seems to induce a specific imprint that favors tolerance. The result is a monocyte/macrophage pool primed to suppress rather than fight new threats.

### 4.3 Impaired antigen presentation

Finally, TB interferes with antigen-presenting cells. Mycobacterium tuberculosis expresses factors that blunt dendritic cell (DC) function. For instance, the bacterial protease Hip1 impairs DC cytokine secretion and maturation (73). DCs infected with Mtb exhibit reduced IL-12 production and lower expression of MHC-II and costimulatory markers (73). *In vivo*, Mtb can trap DCs in tissues, preventing them from reaching lymph nodes to prime T cells (74, 75). The lasting consequence may be a repertoire of memory T cells that is incomplete or skewed. Although some DC may recover following TB treatment, persistent immune alterations could impair their ability to efficiently present tumor neoantigens, potentially weakening anti-tumor immune surveillance.

**Figure 4:**
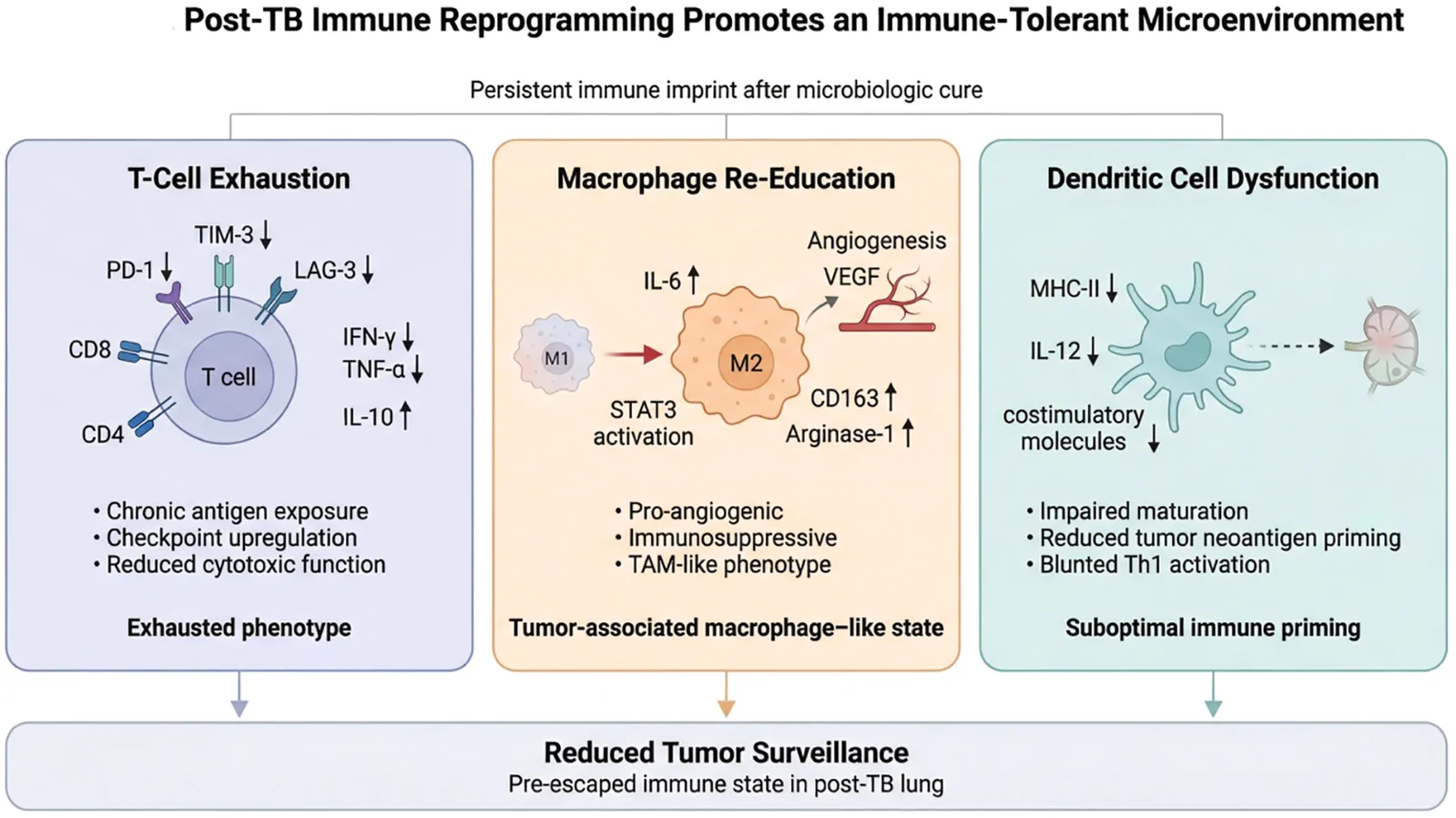
Tuberculosis induces durable immune reprogramming that weakens tumor surveillance. Chronic antigen exposure during TB promotes T-cell exhaustion characterized by PD-1, TIM-3, and LAG-3 upregulation, increased IL-10 production, and reduced effector cytokines. Macrophages are skewed toward an M2-like, tumor-associated phenotype marked by IL-6/STAT3 signaling and CD163 expression, supporting angiogenesis and immune suppression. Dendritic cell maturation and antigen presentation are impaired, limiting effective priming of anti-tumor T cells. Collectively, these alterations establish a pre-escaped immune microenvironment that may facilitate malignant transformation. Created with Biorender.com.

Taken together, TB does more than transiently suppress immunity; it durably reshapes the pulmonary immune landscape (**Figure 4**). The lung becomes populated by exhausted T cells, TAM-like macrophages, and underpowered DCs, a constellation resembling the immune-tolerant tumor microenvironment (73, 76, 77). In effect, TB creates a “pre-escaped” immune state: one that a budding cancer could exploit, lowering the threshold for malignant progression.

## 5 Chronic inflammation, DNA damage, and oncogenic signaling

The changes above create chronic inflammation, which directly fuels carcinogenesis through classical mechanisms. TB-induced inflammation floods the lung with reactive oxygen and nitrogen species (ROS/RNS) from immune cells. Neutrophils, macrophages and damaged epithelium all release ROS and nitric oxide, which can cause DNA strand breaks and base modifications in surrounding cells (78, 79). This genotoxic milieu is compounded by repeated tissue injury: epithelial cells in fibrotic or bronchiectatic regions must proliferate often to regenerate barriers, increasing the chance of replication errors. Inflammatory cytokines further exacerbate DNA damage by promoting error-prone repair and fostering a microenvironment where mutations accumulate.

Inflammation also triggers oncogenic signaling pathways. NF-κB and STAT3 are central mediators of inflammation-driven cancer. Pro-inflammatory cytokines such as IL-1, TNF-α and IL-6, abundantly produced in TB granulomas and scars (51, 80), activate NF-κB and STAT3 in lung epithelial cells. Activated NF-κB induces anti-apoptotic and proliferative genes; STAT3 drives proliferation and EMT-promoting transcriptional programs. Chronic NF-κB/STAT3 signaling establishes a vicious cycle: it enhances survival of damaged cells and further increases pro-tumor cytokines (80). Other pathways are also involved: Wnt/β-catenin signaling, which is co-opted by both Mtb infection and tumors, may become dysregulated. (Indeed, Mtb directly engages Wnt pathways in macrophages (81), though the downstream effects on lung epithelium warrant more study.) Chronic inflammation in TB thus converges on the same pathways that drive lung tumorigenesis in other settings (smoking, COPD, fibrosis). The persistent inflammatory milieu of the post-tuberculosis lung is sustained by microenvironment shifts toward driven cellular senescence and matrix structural remodeling (82, 83). Recent single cell and spatial transcriptomic profiling has revealed that TB-induced myofibroblasts (MMP1^+^CXCL5^+^) persist long after microbiological clearance, acting as chronic drivers of structural pathology by continuously remodeling the local extracellular matrix and anchoring a localized loop of fibrotic distortion (82). This aberrant mesenchymal activity operates in tandem with widespread cellular senescence within the vascular niche. Specifically, chronic mycobacterial insults trigger a profound downregulation of homeostatic survival pathways, such as FOXO3 signaling, driving endothelial cells into a senescent state (83). These senescent endothelial cells actively secrete a robust senescence-associated secretory phenotype (SASP) that propagates NF-κB-dependent thromboinflammation. Together, this intersection of persistent myofibroblast activity and SASP-mediated inflammation establishes a highly permissive, pro-tumorigenic tissue niche, providing both the architectural matrix and the chronic inflammatory stimuli that facilitate pre-malignant clonal expansion.

One dramatic outcome of inflammation is epithelial–mesenchymal transition (EMT) (84). Cytokines like TGF-β and TNF-α, elevated in healed TB lesions (85, 86), can induce epithelial cells to adopt mesenchymal, migratory phenotypes. EMT not only enables invasion, but also endows cells with stem-like properties and resistance to apoptosis. Over many years, repeated cycles of cytokine exposure and repair may push some epithelial clones into a quasi-malignant EMT state. At the very least, this sets the stage for the “classic” multistage carcinogenesis: DNA damage, mutational activation of oncogenes (e.g. EGFR, which is reportedly enriched in post-TB cancer (24, 25)), and loss of tumor suppressors.

**Figure 5:**
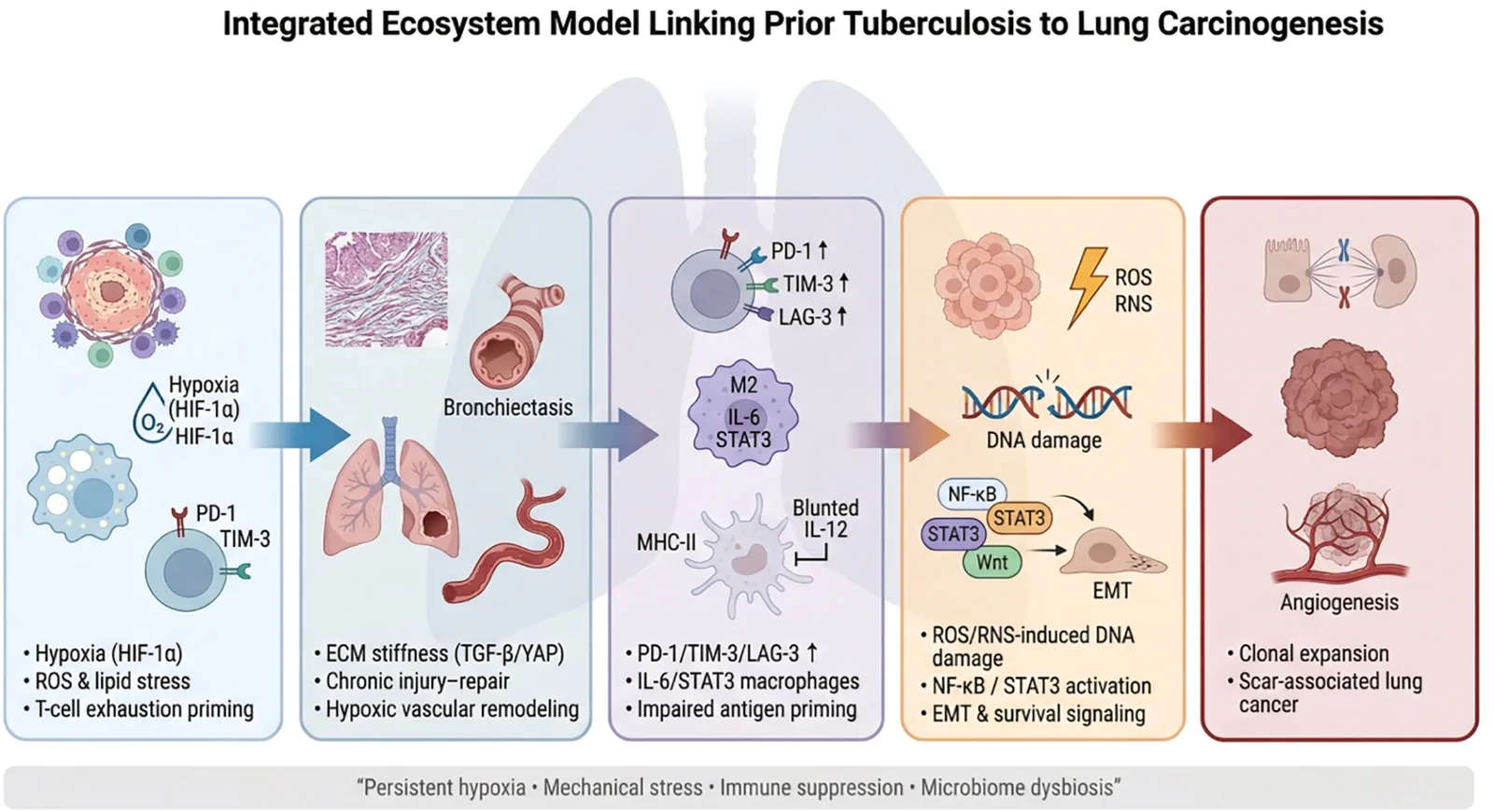
Integrated ecosystem model linking prior tuberculosis to lung carcinogenesis. Tuberculosis granulomas generate hypoxia, oxidative stress, and T-cell exhaustion. Even after microbiologic cure, persistent structural remodeling, including fibrosis, cavitation, bronchiectasis, and vascular abnormalities creates a mechanically and metabolically altered lung scaffold. Concurrent immune reprogramming characterized by checkpoint upregulation, macrophage polarization, and impaired antigen presentation weakens tumor surveillance. Chronic inflammation and oncogenic signaling (NF-κB, STAT3, Wnt, EMT pathways) promote DNA damage and clonal expansion, ultimately culminating in scar-associated lung cancer. This model conceptualizes TB as a long-term architect of a tumor-permissive lung ecosystem. Created with Biorender.com.

In short, the chronic inflammatory legacy of TB in the lung is a fertile ground for carcinogenesis: excessive ROS damage DNA, repeated regeneration increases replication stress, and sustained NF-κB/STAT3/Wnt signaling drives proliferation and EMT (80, 87–89). These are the hallmarks of inflammation-associated cancer (90, 91). The pieces fit: TB leaves behind a setting of chronic immunopathology that mechanistically aligns with known tumor-promoting processes, transforming the lung into a tumor-permissive ecosystem (**Figure 5****).**

## 6 The post-TB lung microbiome: an emerging hypothesis

The lung microbiome has emerged as an important regulator of pulmonary immune homeostasis and is increasingly recognized as a contributor to lung cancer biology (92). Tsay and colleagues demonstrated that enrichment of lower-airway Veillonella, Prevotella, and Streptococcus in early-stage NSCLC is associated with activation of PI3K/ERK signaling in airway epithelial cells, while lower airway dysbiosis is associated with poorer clinical outcomes (93, 94). In addition, both the lung and gut microbiome have been implicated in modulating responses to immune checkpoint inhibitors, with Akkermansia muciniphila being associated with improved anti-PD-1 efficacy in NSCLC (95, 96).These observations highlight an important interaction between microbial communities, host immunity, and tumor biology, although they are not specific to tuberculosis.

Whether pulmonary tuberculosis induces similar long-term microbial alterations remains largely unknown. Nevertheless, post-TB structural lung disease, particularly bronchiectasis, is associated with persistent bacterial colonization by organisms including Pseudomonas, Haemophilus, Staphylococcus, and nontuberculous mycobacteria (57, 58). Chronic colonization may perpetuate neutrophilic airway inflammation and oxidative stress, processes that are well established in bronchiectasis and could contribute to persistent epithelial injury (47, 57). However, direct evidence linking post-TB microbial dysbiosis to lung carcinogenesis is currently lacking.

**Figure 6:**
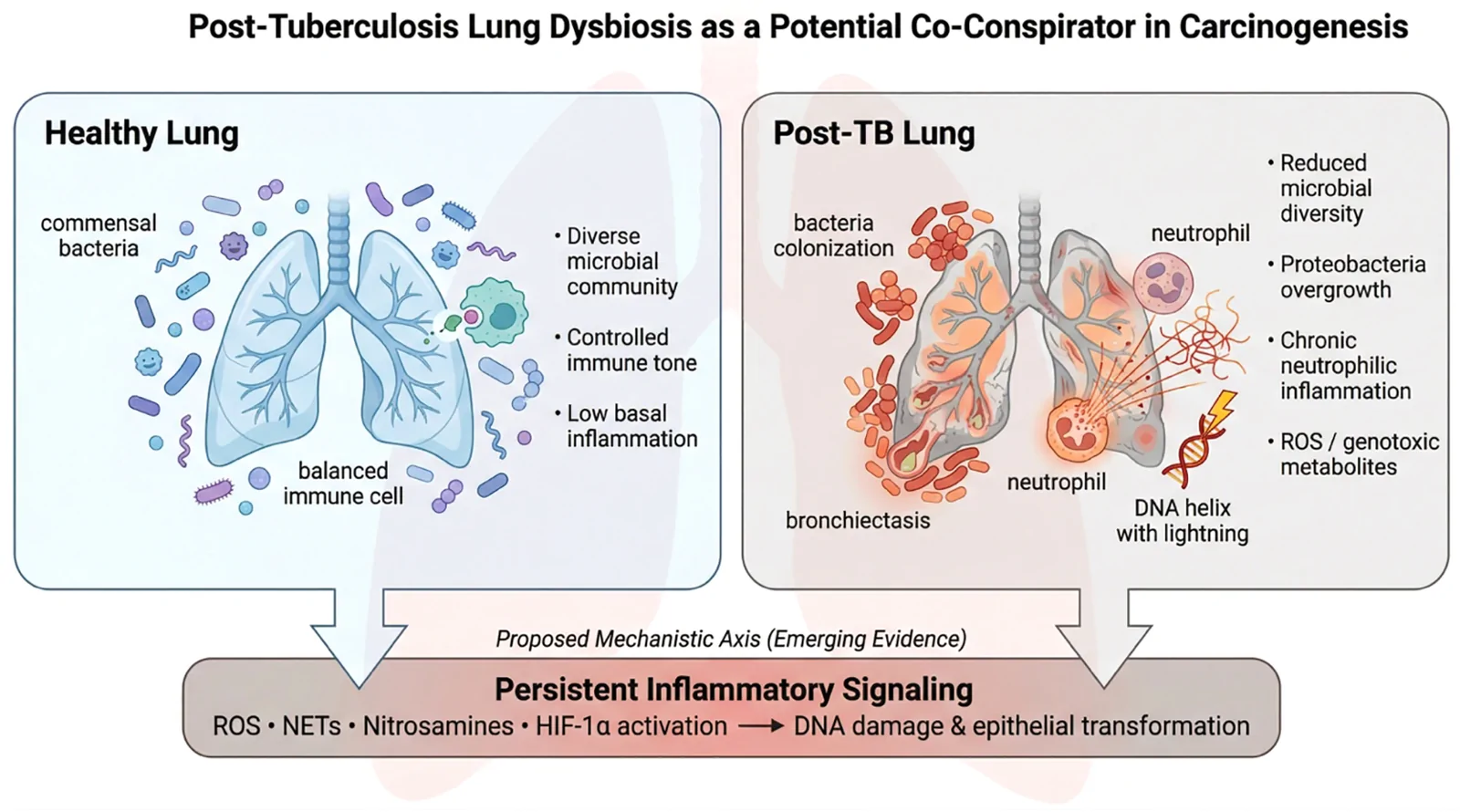
Post-tuberculosis lung dysbiosis may contribute to carcinogenesis. In healthy lungs, a balanced and diverse microbiome supports immune homeostasis and low basal inflammation. In contrast, post-TB structural damage, including fibrosis and bronchiectasis, fosters microbial dysbiosis characterized by reduced diversity and overgrowth of pro-inflammatory taxa. Chronic neutrophilic inflammation, reactive oxygen species production, and genotoxic microbial metabolites may sustain epithelial DNA damage and inflammatory signaling, potentially contributing to malignant transformation. This axis remains an emerging hypothesis requiring further investigation. Created with Biorender.com.

As illustrated in **Figure 6**, we propose that TB-induced structural remodeling may alter the pulmonary microbial ecosystem, thereby sustaining chronic inflammation and influencing epithelial–immune interactions (We are not aware of direct citations of TB→microbiome→lung cancer, highlighting this as an emergent hypothesis.). Future longitudinal studies integrating metagenomic sequencing, microbial metabolomics, and experimental models will be required to determine whether post-TB dysbiosis contributes to lung carcinogenesis and whether specific microbial signatures can be exploited for risk stratification or therapeutic intervention.

## 7 Reverse causality, surveillance bias, and residual confounding

Any proposed causal link between prior tuberculosis (TB) and lung cancer must grapple seriously with alternative explanations. Observational associations particularly those derived from administrative databases and retrospective cohorts are vulnerable to reverse causality, surveillance bias, and residual confounding. A careful appraisal of these issues is essential before attributing the excess lung cancer risk in TB survivors to biological imprinting alone.

### 7.1 Reverse causality: was the cancer already there?

One of the most frequently raised concerns is reverse causality. Lung cancer, particularly when centrally located or cavitary, can radiographically and clinically mimic pulmonary TB. Symptoms such as chronic cough, weight loss, hemoptysis, and constitutional decline overlap substantially. In high TB-burden settings, clinicians may initiate anti-tuberculous therapy empirically in patients with suggestive imaging, especially when microbiological confirmation is lacking. In such cases, a cancer present at baseline may only become apparent months later, creating the illusion that TB preceded malignancy.

The temporal pattern observed in epidemiologic studies lends some support to this concern. Several meta-analyses have shown that the risk of lung cancer is highest within the first one to two years following TB diagnosis. *Luczynski* et al. reported a markedly elevated standardized incidence ratio in the early post-TB period, with attenuation over time (9). Similarly, *Cabrera-Sanchez* et al. found that hazard ratios were most pronounced in the first two years after TB treatment completion, then declined but remained modestly elevated thereafter (10). Earlier work by *Liang* et al. also emphasized the clustering of lung cancer diagnoses soon after TB, suggesting that misclassification or pre-existing malignancy could partly explain early excess risk (18).

However, reverse causality is unlikely to fully account for the association. First, many contemporary cohort studies exclude cancers diagnosed within the first 6–12 months after TB diagnosis and still observe a persistent elevation in risk (11, 97). Second, long-term follow-up extending beyond five years continues to demonstrate increased incidence compared with matched controls (98). These observations suggest that while early cases may reflect occult malignancy, a sustained biological effect may operate beyond the window of diagnostic confusion.

### 7.2 Surveillance bias: the consequence of increased imaging

TB survivors frequently undergo repeated imaging during treatment and follow-up, including chest radiographs and, in some settings, computed tomography. This heightened surveillance may increase the likelihood of detecting asymptomatic or early-stage lung cancers compared with the general population.

Surveillance bias would predict an apparent excess of early-stage tumors diagnosed soon after TB treatment, potentially inflating incidence estimates. Indeed, studies have noted a peak in lung cancer diagnoses in the first year following TB (9, 18), which could reflect more intensive clinical contact and imaging rather than true carcinogenesis.

Yet, several factors argue against surveillance bias as the sole explanation. First, the increased risk is observed even in large population-based datasets where follow-up imaging after TB is not systematically performed beyond treatment completion (11). Second, the magnitude of risk often exceeds what would be expected from detection bias alone, particularly when hazard ratios approach two- to three-fold. Third, if surveillance were the dominant factor, one might expect a predominance of early-stage cancers with improved survival; however, many studies do not report dramatically better stage distribution among TB survivors compared with controls.

Importantly, the persistence of elevated risk years after TB treatment well beyond the period of routine follow-up suggests that surveillance bias may amplify early incidence but cannot fully explain long-term associations (10, 98).

### 7.3 Residual confounding: smoking, COPD, and socioeconomic factors

Smoking remains the most powerful risk factor for lung cancer, and TB is more prevalent among smokers. Even when studies adjust for smoking status, residual confounding may persist due to incomplete quantification (e.g., pack-years, duration, cessation timing). The Korean population-based study by *Moon* et al. adjusted for smoking and chronic obstructive pulmonary disease (COPD) yet still found TB to be an independent risk factor (11). Nonetheless, misclassification of smoking intensity or unmeasured environmental exposures (such as biomass fuel smoke or occupational silica exposure) may partially contribute.

COPD represents another potential confounder. Post-TB lung disease often manifests with airflow limitation resembling COPD (56). Since COPD independently increases lung cancer risk, disentangling the contribution of TB from that of chronic airflow obstruction can be challenging. Some analyses attempt to stratify by COPD status and continue to observe excess risk among TB survivors (11), but residual overlap likely remains.

Socioeconomic disadvantage also intersects with both TB and lung cancer. Crowded living conditions, poor nutrition, environmental pollutants, and limited healthcare access may influence both diseases (99). While multivariable adjustments reduce confounding, no observational study can eliminate it entirely.

An important conceptual distinction should be made between confounding and mediation. Chronic inflammation, structural lung damage, and immune dysregulation may be viewed as downstream consequences of TB rather than independent confounders. For example, post-TB bronchiectasis and fibrosis are not external exposures but biological sequelae of infection (56). If these conditions increase cancer risk, they may represent mechanistic intermediates rather than confounders to be adjusted away.

Large pooled analyses, such as the International Lung Cancer Consortium study by *Brenner* et al., have shown that prior lung diseases including TB are associated with increased lung cancer risk even after accounting for smoking (100). These findings support the idea that chronic inflammatory remodeling of lung tissue, regardless of etiology, contributes to carcinogenesis.

Taken together, reverse causality likely explains a fraction of early cancer diagnoses after TB, and surveillance bias may inflate short-term incidence. Residual confounding particularly from smoking and COPD cannot be entirely excluded. However, several consistent observations argue for a genuine biological association:

- The risk persists beyond the immediate post-diagnosis period (10, 98).
- The association is observed in never-smokers (9, 11).
- Adjusted hazard ratios remain elevated after controlling for major confounders (11, 97).
- The magnitude of risk aligns with mechanistic plausibility based on chronic inflammation and fibrotic remodeling (51, 53, 90).

Thus, while epidemiologic caution is warranted, the convergence of temporal patterns, adjustment analyses, and biological plausibility supports the interpretation that TB is more than a coincidental marker of risk. It may act as a permissive architect of a pro-carcinogenic lung environment, even if part of the early signal reflects diagnostic and surveillance artifacts.

A nuanced interpretation is therefore appropriate: early excess risk may partly reflect misdiagnosis and intensified clinical scrutiny, whereas sustained long-term elevation likely reflects structural and immune remodeling that endures beyond microbiologic cure. Recognizing and disentangling these layers strengthens, rather than weakens, the argument that prior TB represents a meaningful and biologically credible modifier of lung cancer risk.

## 8 Clinical implications: from surveillance to prevention

The recognition that TB can be a premalignant risk state has practical implications. Clinicians and public health experts should consider tb survivors as a high-risk group for lung cancer, warranting tailored strategies. **Table 2** shows immune alterations in post-tb and its impact on tumor surveillance whereby these alterations blunt anti-tumor immunity. for example, tb drives pd-1^+^ exhausted t cells (76) and m2-like macrophages (77), while impairing dc function (73). we outline possible approaches in risk stratification, surveillance and prevention.

**Table 2:** Immune alterations post-tb and impact on tumor surveillance.

Immune component	Normal anti-tumor role	Post-TB alteration	Tumor-related consequence	References
CD8^+^/CD4^+^ T cells	Cytotoxic killing; IFN-γ/TNF-α release	Upregulated PD-1, TIM-3, LAG-3; increased IL-10	Exhausted phenotype; reduced tumor cell killing and surveillance	(43, 63, 101)
Macrophages	Phagocytosis; pro-inflammatory (M1)	Shift toward M2-like/TAM phenotype; high CD163, STAT3	Immunosuppressive, pro-angiogenic; secrete growth factors (IL-6, VEGF)	(77)
Dendritic cells	Antigen presentation, IL-12 production	Impaired maturation: low MHC-II/IL-12 (Hip1-mediated)	Suboptimal T cell priming to tumor antigens; blunted Th1 immunity	(73)
Neutrophils	Early defense; ROS burst	Chronic recruitment via IL-8, IL-1β in bronchiectasis	Excess ROS/DNA damage (NETs, elastase) but paradoxically also pro-tumor (angiogenesis)	(47)
NK/NKT cells	Killing of transformed cells	Possibly reduced activity in chronic inflammation (data limited)	Less innate tumor surveillance	(102)
Checkpoints (PD-1/PD-L1, CTLA-4)	Normally low (activate T cells)	Upregulated on TB-exposed T cells, macrophages and even lung epithelium	Enhanced inhibitory signaling; tumors can exploit these pathways	(73, 76)

### 8.1 Risk stratification in TB survivors

Not all TB survivors carry equal risk. Certain features of post-TB lung disease likely mark higher cancer risk. For instance, extensive fibrotic scarring on imaging is probably more dangerous than mild residual nodularity. Patients with cavitary or bronchiectatic changes may have more persistent inflammation and thus higher risk. Clinical factors such as older age at TB, male sex, or persistent symptoms (chronic cough, hemoptysis) might also identify higher-risk individuals. Additionally, immune biomarkers could eventually help stratify risk. For example, elevated levels of systemic inflammatory markers (IL-6, C-reactive protein) or persistent immune exhaustion markers (circulating PD-1^+^ T cells) in a TB survivor might hint at continued tissue remodeling. To date there are no validated “TB-survivor scores” for cancer risk, but research should aim to integrate radiologic (extent of fibrosis/cavitation (103), spirometric (degree of obstruction), and immunologic parameters.

### 8.2 Imaging and biomarker opportunities

Given this risk, clinicians might consider enhanced surveillance for lung cancer in TB survivors. One approach is judicious use of imaging. Low-dose CT scans, now standard for high-risk smokers (104), could be considered for older TB survivors with extensive lung damage. For example, a patient aged 60+ with bilateral fibrotic scars might benefit from periodic CT screening for early neoplasms in scarred regions. Radiologists should also be vigilant: new nodules emerging at scar sites or around old cavities may represent primary cancers rather than post-inflammatory changes. Quantitative imaging measuring fibrosis volume or texture may further stratify risk in future.

On the biomarker side, there is intriguing potential. Circulating markers of immune exhaustion or inflammation could flag patients needing closer follow-up. For instance, persistently elevated plasma IL-6 or IFN-γ/PD-1 gene signatures (as used in some immunotherapy predictors) might indicate an ongoing pro-tumor milieu (105). Immune profiling of peripheral blood (e.g. high PD-1^+^CD8^+^ T cell fraction (76)) might correlate with risk, although this is speculative. Urinary or breath biomarkers (like volatile organic compounds linked to lung cancer) could eventually play a role as well. Overall, there is a rich research opportunity to identify predictive markers specific to the post-TB state.

### 8.3 Therapeutic and preventive concepts

Ultimately, we may strive not just to detect cancer early, but to mitigate the risk. Potential strategies include:

#### 8.3.1 Anti-fibrotic therapy

Drugs like pirfenidone or nintedanib, which slow fibrosis in idiopathic pulmonary fibrosis (106, 107), might be repurposed for severe PTLD (post-TB lung disease) to reduce scar formation. Inhibiting TGF-β signaling in high-risk patients could theoretically lessen mechanotransduction-driven tumor promotion. Important caveats must be made explicit. First, both pirfenidone and nintedanib have been licensed and tested almost exclusively in IPF and progressive-pulmonary-fibrosis phenotypes; their efficacy and safety in post-TB fibrosis are entirely untested, and the focal, granuloma-derived architecture of TB scarring may respond differently from the diffuse fibroblastic-foci pattern of IPF. Second, and more critically, TGF-β is a central regulator of granuloma containment of *M. tuberculosis* and of regulatory T-cell-mediated tolerance; therapeutic TGF-β inhibition in a post-TB patient carries a theoretical but plausible risk of latent TB reactivation, analogous to the well-documented reactivation risk seen with TNF-α blockers. Any future trial of pirfenidone, nintedanib, or selective TGF-β pathway inhibitors in TB survivors must therefore include rigorous pre-treatment screening for latent TB infection (IGRA), consideration of preventive TB therapy, and prospective monitoring for reactivation. The same caveats apply to repurposing of tocilizumab and other IL-6/JAK–STAT inhibitors discussed below.

#### 8.3.2 Immune re-invigoration

The parallels between TB ‘exhausted’ lungs and cancer suggest testing immune checkpoint modulators carefully. For example, trials of PD-1 blockade in TB survivors (with no active infection) could possibly rejuvenate anti-tumor immunity. However, caution is needed since anti-PD-1 can cause reactivation in latent TB infection (LTBI) and drastically worsen the disease in active TB patients (76). This concern is no longer theoretical: case series and pharmacovigilance reports of pembrolizumab- and nivolumab-treated NSCLC patients have documented active TB reactivation, in some cases presenting as immune-related adverse events that radiographically and clinically mimic checkpoint pneumonitis. Published case reviews (e.g., *Anastasopoulou* et al. (108); *Langan* et al. (109)) collectively describe several dozen confirmed cases, with median onset 3–6 months after PD-1/PD-L1 blockade initiation, and emphasize the need for baseline IGRA screening, judicious use of preventive isoniazid in IGRA-positive patients, and a low threshold to send mycobacterial cultures when new cavitary or upper-lobe infiltrates appear during checkpoint therapy. These data shift the recommendation from a generic caution to an actionable, evidence-based screening pathway. Perhaps a safer approach is targeting IL-6/STAT3 with agents like tocilizumab or STAT3 inhibitors, to counter the chronic immunosuppressive signaling present post-TB.

#### 8.3.3 Antimicrobial control

Managing chronic lung infections (e.g. with inhaled antibiotics for bronchiectasis) could reduce pro-carcinogenic inflammation. Similarly, emerging therapies targeting the lung microbiome (probiotics or selective bacteriophages) might be explored.

#### 8.3.4 Lifestyle and chemoprevention

Ensuring TB survivors avoid smoking and environmental exposures is crucial (110, 111). Low-dose aspirin or metformin, which have shown modest lung cancer prevention effects (112, 113), could be considered especially in those with heavy residual inflammation (although trials are needed). We must emphasize that no chemoprevention trial of aspirin or metformin has been conducted in TB survivors specifically. The signals supporting these agents derive from general-population observational studies and from cohorts not enriched for prior TB. We therefore frame both as strictly hypothesis-generating in the post-TB context, with no current evidence to recommend their use for lung-cancer chemoprevention in TB survivors. Any future trial in this population would also need to address the metformin–antituberculosis-drug interaction question (metformin’s host-directed antimycobacterial effects, and possible interactions with isoniazid hepatotoxicity profile) as part of trial design.

Tailored screening protocols: In TB-endemic regions, adding TB history as a risk factor in lung cancer screening guidelines may be warranted. For example, a 55-year-old non-smoker with fibrotic TB scars might still qualify for CT screening even if not meeting traditional criteria.

**Figure 7:**
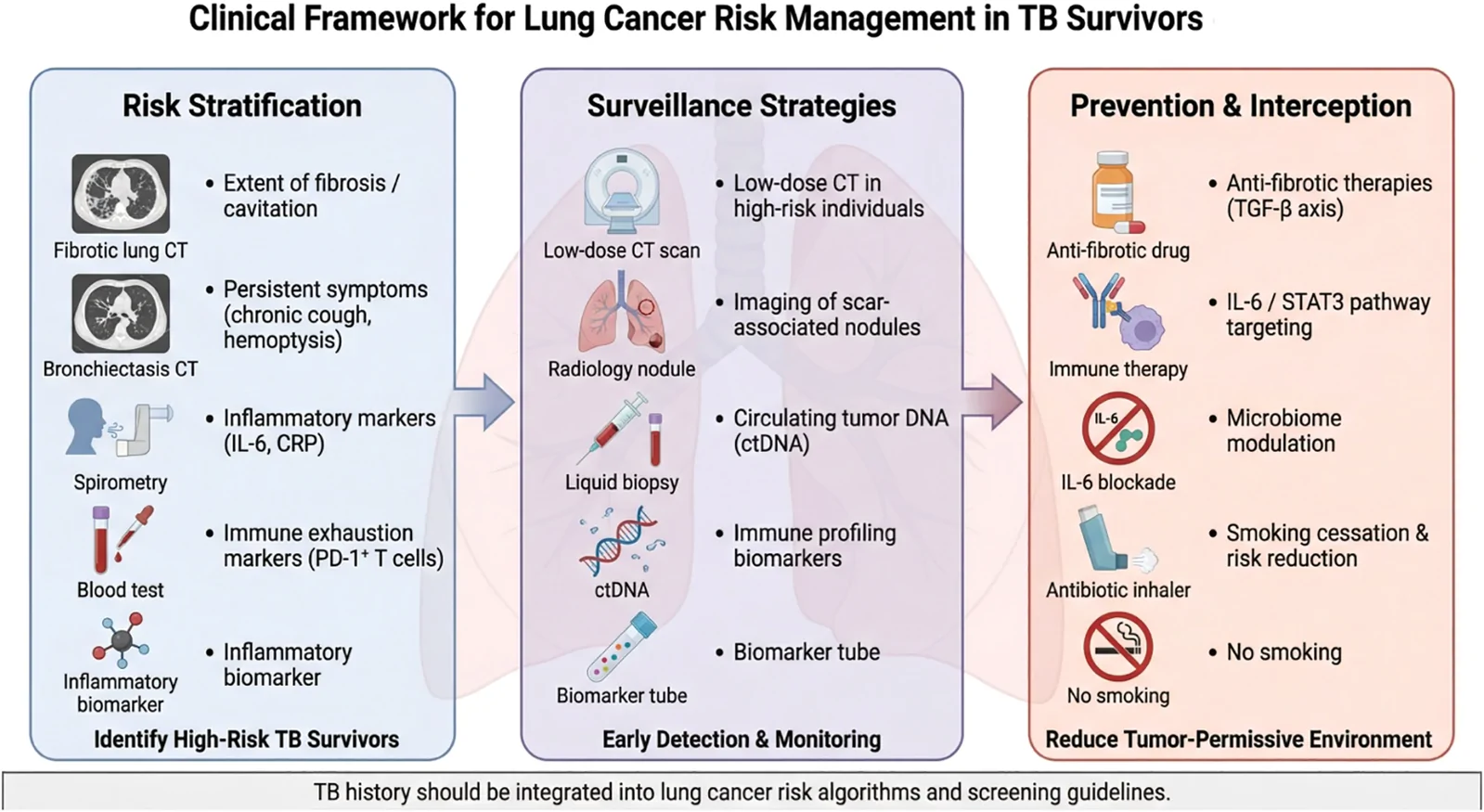
Translational framework for lung cancer risk management in tuberculosis survivors. Structural and immune features of post-TB lung disease may allow risk stratification based on fibrosis burden, persistent inflammation, and immune exhaustion markers. High-risk individuals could benefit from tailored surveillance using low-dose CT and emerging biomarkers such as circulating tumor DNA or immune profiling. Preventive strategies, including anti-fibrotic therapies, modulation of inflammatory signaling pathways, microbiome-directed interventions, and risk reduction measures, may help mitigate the tumor-permissive microenvironment. Integrating TB history into lung cancer risk algorithms may improve early detection and outcomes. Created with Biorender.com.

These ideas require clinical trials, but they illustrate the range of possibilities when TB is viewed as a chronic modifier of cancer risk (**Figure 7****).**

## 9 Knowledge gaps and future directions

Despite growing recognition of the link between tuberculosis (TB) and lung carcinogenesis, substantial knowledge gaps remain that limit both mechanistic understanding and clinical translation. Foremost among these is the lack of longitudinal immune profiling in TB survivors. Prospective studies tracking immune cell phenotypes over extended periods are needed to determine the persistence and functional relevance of exhausted or reprogrammed immune states, such as PD-1^high T cells or CD163^high macrophages, and whether these correlate with subsequent cancer development. Such efforts could be enabled by serial bronchoscopic sampling, peripheral immune profiling, and advanced imaging modalities capable of capturing dynamic immune activity within post-TB lung tissue.

At the tissue level, high-resolution characterization of post-TB lung disease (PTLD) remains incomplete. Emerging technologies such as single-cell RNA sequencing and spatial transcriptomics offer the opportunity to define persistent cellular niches within fibrotic or structurally remodeled lung. These approaches may reveal, for example, the presence of lung-resident exhausted T-cell clones, pathogenic fibroblast subsets, or epithelial populations exhibiting pre-malignant transcriptional programs. Integrating these datasets with histopathology and imaging could establish a spatially resolved atlas of the proposed post-TB pre-malignant niche.

As immune checkpoint inhibitors become standard of care in lung cancer, understanding how prior TB shapes immunotherapy response is increasingly important. It remains unclear whether TB-associated scarring and immune reprogramming enhance or impair checkpoint inhibitor efficacy, or whether they predispose patients to altered toxicity profiles. Conversely, checkpoint blockade may disrupt immune containment of latent Mycobacterium tuberculosis, raising the risk of reactivation in susceptible individuals. These bidirectional interactions between TB and immunotherapy represent a critical and underexplored area of translational research.

From a clinical perspective, evidence-based screening strategies for lung cancer in TB survivors are lacking. While epidemiologic data suggest elevated risk, optimal screening criteria including; which patients to screen, at what age, and with what frequency have not been defined. Cost-effectiveness analyses and risk-stratified screening models will be essential to inform guideline development, particularly in TB-endemic settings.

The role of the lung microbiome in post-TB carcinogenesis also remains poorly understood. Comprehensive metagenomic profiling of sputum and bronchoalveolar lavage samples may identify persistent microbial communities or metabolic signatures that contribute to chronic inflammation or carcinogenic signaling. Coupling microbiome studies with experimental models could help establish causal relationships between microbial dysbiosis and tumor development.

At the mechanistic level, many of the pathways proposed in this review, including YAP/TAZ-mediated mechanotransduction, IL-6/STAT3 signaling, and Wnt-driven epithelial plasticity, remain incompletely validated in TB-specific contexts. Functional studies are needed to determine whether targeting these pathways can modify disease progression. For example, it is unknown whether inhibition of YAP/TAZ signaling attenuates tumorigenesis in fibrotic TB models, or whether IL-6 blockade alters the evolution of post-TB scar tissue. Bridging these mechanistic insights to therapeutic intervention represents a key step toward clinical translation.

Perhaps the most significant limitation in the field is the lack of animal models that reproduce the long-term consequences of pulmonary tuberculosis after microbiological cure. Although *Nalbandian* et al. demonstrated that chronic Mycobacterium tuberculosis infection can promote lung carcinogenesis in a mouse model (114), this model primarily reflects the effects of persistent infection rather than the post treatment state observed in Tb survivors (114). Consequently, there is still no experimental model that fully captures the persistent structural remodeling, immune dysregulation and chronic inflammation changes that characterize the post-TB lung and are thought to contribute to subsequent lung carcinogenesis. Addressing this gap is essential for establishing causality and testing interventions. A feasible experimental framework would involve aerosol infection of mice (e.g., BALB/c or C57BL/6) with M. tuberculosis, followed by antimicrobial treatment to achieve microbiologic cure, and subsequent exposure to a low-dose carcinogenic stimulus. Longitudinal follow-up incorporating imaging, histopathology, and molecular analyses, including single-cell sequencing and genomic profiling could determine whether prior TB enhances susceptibility to tumorigenesis. Parallel non-human primate models, incorporating serial PET–CT imaging and bronchoscopic sampling, would provide a translational platform for validating human-relevant mechanisms and testing preventive or therapeutic strategies.

Addressing these challenges will require close collaboration across disciplines, including TB research, pulmonology, oncology, immunology, and systems biology. Conceptually, this effort aligns with a broader shift toward recognizing TB as a chronic disease with long-term sequelae, analogous to established infection-to-cancer paradigms such as hepatitis-associated hepatocellular carcinoma or Helicobacter pylori–associated gastric cancer. A coordinated focus on post-TB care and long-term outcomes has the potential not only to improve quality of life for TB survivors but also to reduce the burden of lung cancer through earlier detection, risk stratification, and targeted intervention.

## 10 Conclusion

Tuberculosis should be seen not only as an acute infection but as a long-term architect of lung cancer risk (1). The biology of TB granulomas and their aftermath naturally links to the hallmarks of cancer: persistent inflammation, tissue remodeling and immune escape. Cure of the infection often leaves a lung marked by fibrosis, cavitation and a suppressed immune microenvironment. These changes collectively create a fertile “soil” in which malignant clones can thrive. As we move forward, integrating TB history into lung cancer epidemiology, screening and research could greatly benefit public health. Recognizing TB’s lasting imprint, we may better prevent and intercept lung cancer, transforming a paradox into opportunity: the challenge of TB becomes a pathway to novel insights into cancer prevention.
